# First Report of *Echinococcus ortleppi* in Free-Living Wild Boar (*Sus scrofa*) from Portugal

**DOI:** 10.3390/microorganisms9061256

**Published:** 2021-06-09

**Authors:** Teresa Letra Mateus, Maria João Gargaté, Anabela Vilares, Idalina Ferreira, Manuela Rodrigues, Catarina Coelho, Madalena Vieira-Pinto

**Affiliations:** 1 CISAS—Centre for Research and Development in Agrifood Systems and Sustainability, Escola Superior Agrária, Instituto Politécnico de Viana do Castelo, 4900-347 Viana do Castelo, Portugal; 2 Epidemiology Research Unit (EPIUnit), Institute of Public Health, University of Porto, Laboratory for Integrative and Translational Research in Population Health (ITR), 4050-091 Porto, Portugal; 3 National Reference Laboratory of Parasitic and Fungal Infections, Infectious Diseases Department of the National Institute of Health Dr. Ricardo Jorge, Av. Padre Cruz, 1649-016 Lisboa, Portugal; m.joao.gargate@insa.min-saude.pt (M.J.G.); anabela.vilares@insa.min-saude.pt (A.V.); idalina.ferreira@insa.min-saude.pt (I.F.); 4 Departamento de Ciências Veterinárias, Universidade de Trás-os-Montes e Alto Douro (UTAD), 5000-801 Vila Real, Portugal; mmvcr@utad.pt (M.R.); mmvpinto@utad.pt (M.V.-P.); 5 Centro de Ciência Animal e Veterinária, (UTAD), 5000-801 Vila Real, Portugal; ccoelho@esav.ipv.pt

**Keywords:** cystic echinococcosis, *Echinococcus ortleppi*, hunters, one health, wild boar

## Abstract

Cystic echinococcosis (CE) is a zoonosis that is prevalent worldwide. It is considered endemic in Portugal but few studies have been performed on *Echinococcus granulosus sensu lato* and their hosts. In this study, CE cysts are reported for the first time in a free-living wild boar (*Sus scrofa*) in Portugal. The presence of the metacestodes in the liver of the wild boar was identified by morphological features, microscopic examination and molecular analysis. The sequencing of part of the DNA nuclear ribosomal internal transcribed spacer-1 (ITS-1) region revealed a G5 genotype that presently corresponds to *Echinococcus ortleppi*. This is the first report of *E. ortleppi* in Portugal and to the best of the authors’ knowledge, in Europe. These results suggest that wild boar may be a host of CE, namely, crossing the livestock–wildlife interface, which has important public health implications. Wildlife reservoirs must be taken into account as CE hosts and surveillance of game as well as health education for hunters should be implemented using a One Health approach, with implementation of feasible and tailor-made control strategies, namely, proper elimination of byproducts in the field.

## 1. Introduction

Cystic echinococcosis (CE) is a zoonosis that is prevalent worldwide, except for Antarctica, and the burden of infection is particularly significant in the Mediterranean countries and the Iberian Peninsula [1].

This disease is caused by *Echinococcus granulosus sensu lato* (*s.l.*), a tapeworm that infects canids as definitive hosts. The parasite develops in the small intestine, its eggs are excreted through feces and spread into the environment and can be ingested by several intermediate hosts, including domestic and wild mammals who develop the larval cysts or metacestodes, mainly in the liver or in other organs [2]. CE causes economic losses in animal production [3,4], as well as high morbidity and possible death in humans, an aberrant intermediate host. It is considered a neglected and re-emerging disease [3]. In a recent study to assess the most important foodborne parasites in Europe, *E. granulosus s.l.* ranked highest in South-Western Europe [5]. Moreover, this disease is recognized as the most important helminth zoonosis in the Mediterranean area [1].

*E. granulosus s.l.* is a species complex and there is ongoing discussion about its speciation [6,7], however, Vuitton et al. [8], in agreement with other specialists, recognized five species: *E. granulosus sensu stricto* (*s.s.*) (genotypes G1 and G3), *E. equinus* (G4), *E. ortleppi* (G5), *E. canadensis* (G6–G8 and G10) and *E. felidis*.

Portugal is considered as endemic for this species by the World Health Organization [9]. Knowledge about *E. granulosus s.l.* epidemiology in Portugal is limited with regard to the genotypes and their specificity towards hosts. CE cysts are commonly seen in slaughterhouses but the lack of accurate registry does not allow a clear picture of the presence of the parasite in Portugal. Also, no scientific reports are known of regarding wildlife, especially wild large game. Few studies have been performed on the genetic diversity of *E. granulosus s.l.* [10]. The genotypes have implications for the implementation and efficiency of the control programs [11]. Despite this, very little information is available yet about geographic dispersion, animal and human prevalence and diversity of the hosts of the different strains [12], and the real distribution is underestimated [13]. The aim of this study was to provide a morphological and a molecular characterization of a parasitic cystic neoformation attributable to *E. granulosus s.l.* in one wild boar from Portugal. Our investigation addressed a CE cyst identified as belonging to *E. ortleppi* (G5).

## 2. Materials and Methods

**Figure 1:**
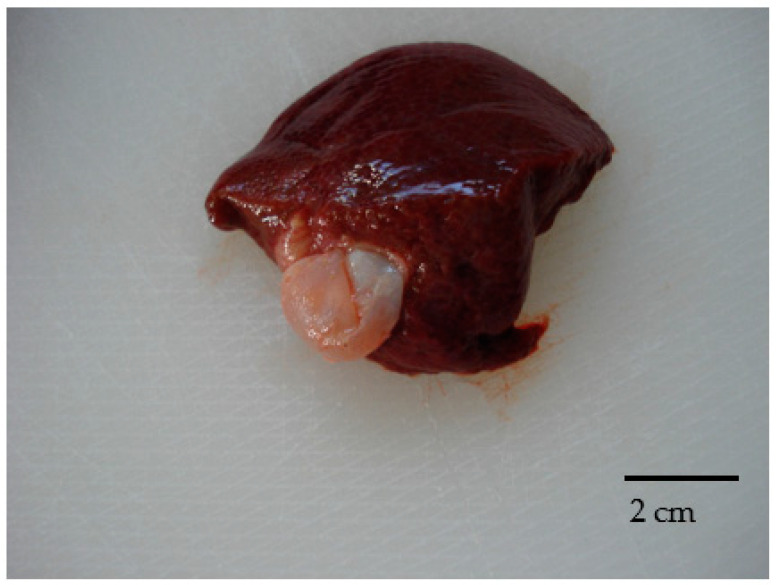
Hepatic cystic lesions.

During the hunting season an adult, free-living wild boar (*Sus scrofa*) was culled in the Northern Portugal. During the initial in loco examination performed by qualified hunters, according to Regulation 853/2004, two intact fluid-filled cystic lesions about 2 cm in diameter (Figure 1), not morphologically suggestive of CE cysts, were detected in the liver.

Nevertheless, the liver was delivered to the Laboratory of the University of Trás-os-Montes and Alto Douro, Vila Real, Portugal, where the parasitological examination by microscopy was performed and the cysts were then prepared for molecular analysis.

A total genomic DNA was extracted from protoscoleces, germinal membranes and liquid from washes [14] in the National Reference Laboratory of Parasitic and Fungal Infections, Infectious Diseases Department of the National Institute of Health Dr. Ricardo Jorge, Lisbon, Portugal. The genomic DNA was not quantified by spectrometry or visualized by an agarose gel because we observed the protoscolex hooks in the samples before the DNA extraction, and in addition, this methodology is performed routinely and validated for the *Echinococcus granulosus* diagnosis in the parasitology laboratory of the National Institute of Health.

For genotyping purposes (performed in the parasitology laboratory of the National Institute of Health, Lisboa, Portugal), DNA samples from the protoscoleces, germinal membranes and hydatic liquid were subjected to different amplifications and a final sequencing of the amplified fragment from DNA nuclear ribosomal internal transcribed spacer-1 (ITS-1) region. The first polymerase chain reaction (PCR) was performed to identify *E. granulosus* G1 (PCR G1), the second to identify G5/6/7 (with no differentiation—g5/6/7 PCR) and the third to identify *E. granulosus* G5 (g5 PCR). The first PCR was performed in a 50 μL volume containing 10 mM Tris–HCl (pH 8.3), 50 mM KCl, 2 mM MgCl_2_, 200 μM of each dNTP, 50 pmoL of each primer (E.g.ss1for.—5′ GTA TTT TGT AAA GTT pool CTA 3′ and E.g.ss1rev.—5′ CTA AAT CAC ATC ATC TTA CAA T 3′) and 2.5 units Ampli-*Taq* Polymerase. Amplification was performed for 40 cycles as follows: denaturation for 30 s at 94 °C, annealing for 1 min at 57 °C and elongation for 40 s at 72 °C. After amplification, 5 μL of the amplification products were detected on 2.5% GelRED (Biotium Inc., Fremont, CA, USA) stained agarose gel electrophoresis. The second PCR assay specifically for *E. granulosus* G5/6/7 was performed in the same conditions as the first PCR with a different primer pair (E.g.cs1for.—5′ ATT TTT AAA ATG TTC GTC CTG 3′ and E.g.cs1rev.—5′ CTA AAT AAT ATC ATA TTA CAA C 3′), as described by Dinkel et al [15]. 

To discriminate between *E. ortleppi* G5 and *E. granulosus* G6/7, a semi-nested PCR specifically for *E. ortleppi* (PCR G5; E.g.cattle.for—5′ ATG GTC CAC CTA TTA TTT TG 3′ and E.g.cs1rev) was used in a second step. The reaction mixture was the same of the previous PCRs, but with different conditions (30 cycles—denaturation for 30 s at 94 °C, annealing for 1 min at 60 °C and elongation for 30 s at 72 °C). The PCR products were visualized in GelRED (Biotium Inc., Fremont, CA, USA) stained 2.5% agarose gel electrophoresis, purified with ExoSAP-IT PCR (Thermofisher, Waltham, MA, USA) according to the manufacturer’s protocol and sequenced with an ABI 3130xl Genetic Analyzer (Applied Biosystems, Foster City, CA, USA). Data were analyzed with Gene Mapper (version 3.7; Applied Biosystems) and a consensus sequence was generated by MEGA5 software (https://www.thermofisher.com/order/catalog/product/4370784#/4370784, accessed on 6 June 2021) [16]. Blast (https://blast.ncbi.nlm.nih.gov/Blast.cgi, accessed on 6 June 2021) was performed to identify the genotypes by comparing the obtained sequence with the ones from reference strains available in GenBank (AY462127, AY462128, AF297617, ECCMTZB, L49456, AJ237778, AJ237779).

## 3. Results and Discussion

**Figure 2:**
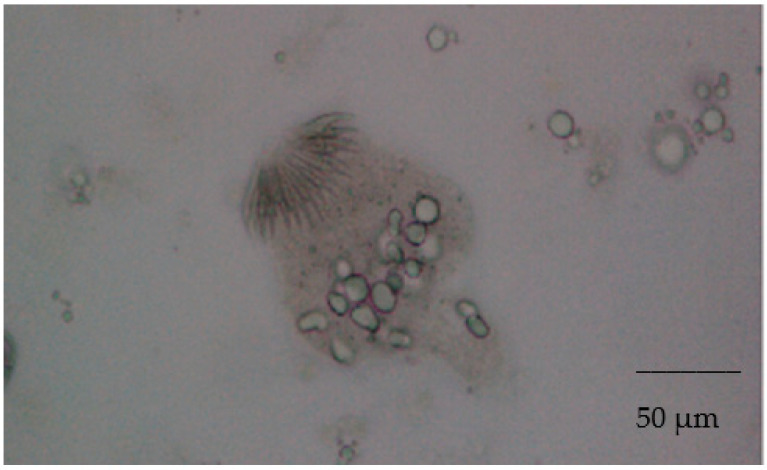
Protoscolex (optical microscope, 40×).

**Figure 3:**
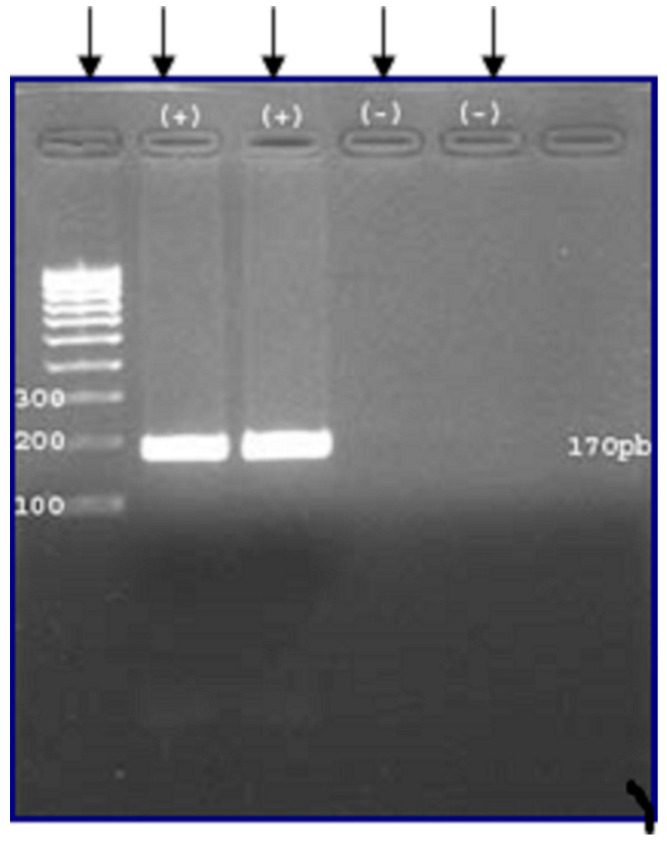
Agarose gel 2.5% electrophoresis of PCR products—from left to right: 100 bp DNA Ladder (first left column); PCR products of 170 pb *E. ortleppi* G5 (second and third columns); negative controls (fourth and fifth columns).

From the liver lesions, the parasitological examination by microscope evidenced protoscoleces (Figure 2) and the PCR results (Figure 3) confirmed the *Echinococcus* spp.

Previous studies in Portugal identified the parasite in sheep and cattle [10,17], wolves [18] and dogs [19]. Regarding the hepatic location of the CE cysts in wild boar, similar results were obtained by Sgroi et al. [20], in contrast to others studies where wild boar lungs were more often found infected [21,22].

The assembled sequence length showed 100% homology in full coverage with the sequence of *Echinococcus ortleppi* genotype 5 (G5) (GenBank ac. number AY462127) that presently corresponds to *E. ortleppi* (Lopez-Neyra and Soler Planas, 1943) [8,23]. Previous studies in Portugal identified *E. granulosus s.s.* [17,19] and *E. canadensis* [10,18], but not in wild boars. According to the current nomenclature [8], *E. granulosus* G7 is named *Echinococcus canadensis* G7.

CE has already been identified in wild boars from Ukraine (*E. granulosus* G7) [24], Romania (*E. granulosus* G1 and G7) [25], France (*E. granulosus* G6/G7) [26], Turkey (*E. granulosus s.s.*) [27], Italy (*E. granulosus* G1, G3, G7) [20,21,28,29,30], and Spain (*E. granulosus* G1, G7) [22,31], but *E. ortleppi* was not found in any of these cases.

Cattle is the usual *E. ortleppi* intermediate host but other ungulates may be involved [32,33], namely, wild hosts, as these parasites use a predator–prey relationship to spread [2]. Other ruminants such as sheep, goats, camels, deer and buffaloes), pigs, monkeys, crested porcupines and lemurs [15,34,35,36,37,38,39,40] have already been identified as harbouring *E. ortleppi* cysts.

Human cases of this infection [32,41,42,43] are rare, it is not known whether the parasite is infrequent or whether humans are more resistant to this species [44]. Nevertheless, it is known that in rural areas with traditional farms and poor hygiene conditions, there is an increased risk of human CE [45] and this corresponds to the geographical area where the positive-wild boar was hunted. This is particularly worrying from a public health point of view, when it has been found by Mateus et al. [46] that there is limited knowledge about CE among farmers from northern Portugal. Farmers and hunters are linked because in this region farmers are usually also hunters, and the parasite cycle may also cross domestic and wild animals in a livestock–wildlife setting. As Gori et al. [47] suggested, dogs may ingest the offal of hunted wild boar and wolves may ingest the offal of grazing domestic animals. Therefore, more epidemiological studies involving wild and domestic carnivores (with special attention to shepherd and hunting dogs) and ungulates are due to be done, and wildlife reservoirs should be taken into consideration for the management of the disease [21].

The number of wild boars are not only increasing throughout Europe, but they are also the most cosmopolitan wild ungulate in the Iberian Peninsula [48]. Additionally, the results of this study suggest that wild boar can be involved in the epidemiology of *Echinococcus* spp., particularly considering that considerable amounts of viscera may remain in the field and therefore available to dogs and wild carnivore species during the hunting season [31,49]. Incorrect disposal of offal and free-roaming dogs (very common in this rural region) are known to contribute to the transmission of these parasites [50,51]. In Italy, Sgroi et al. [20] reported that hunting dogs are fed wild boar offal as a reward, and this is also a common practice in Portugal.

The present study also underlines the importance of the first in loco examination after the hunting campaign for the detection of gross pathological findings and for the implementation of corrective measures in order to mitigate the zoonotic risk, both in the game meat production chain and in the environment. Therefore, hunters who are well educated on these matters could be important allies if CE is identified in game [20]. The complexity of the life cycle and the diversity of hosts associated with CE calls for the involvement of different specialists and areas, who should cooperate in the One Health perspective [8]. However, it is also important to include context-targeted interventions [51].

As future research, coprological analyses of wild and domestic carnivores from the same region should be done to clarify the epidemiology of the disease in this region. Wild boars may be viewed as sentinel animals with regard to the presence of CE in domestic animals [20].

This report is the first recording of *E. ortleppi* infection in a wild boar in Europe and significantly contributes to our knowledge of the host range and geographical distribution of CE, which has rarely been reported in Portugal. From a One Health perspective there is a pressing need to proceed to a molecular survey including domestic and wild animals and humans in order to see the whole scenario and better understand the epidemiology of CE, as previously highlighted by Tamarozzi et al. [52]: there is also an evident problem in European Mediterranean and Balkan countries in the underreporting of CE infections in both humans and animals. This would also allow control interventions to be prioritized and tailored to prevent CE.

## Data Availability

The data that support the findings of this study are available from the corresponding author, upon request.
